# What to Do in Cases of Poisoning

**Published:** 1889-12

**Authors:** 


*What to do in Cases of Poisoning.* By WILLIAM MURRELL, M.D. Sixth Edition. London: H. K. LEWIS. 1889.

In this pocket-volume no words are wasted. It is essentially practical, and, containing the latest information on the questions of diagnosis and treatment, is just the book for the anxious times which come to the practitioner when least expected.

